# Evaluation of optimum roughage to concentrate ratio in maize stover based complete rations for efficient microbial biomass production using *in vitro* gas production technique

**DOI:** 10.14202/vetworld.2016.611-615

**Published:** 2016-06-19

**Authors:** Y. Ramana Reddy, N. Nalini Kumari, T. Monika, K. Sridhar

**Affiliations:** 1 Department of Animal Nutrition, College of Veterinary Science, Kadapa - 516 360, Andhra Pradesh, India; 2 Department of Animal Nutrition, College of Veterinary Science, Hyderabad - 500 030, Telangana, India

**Keywords:** complete ration, *in vitro* gas technique, maize stover, roughage to concentrate ratio

## Abstract

**Aim:**

A study was undertaken to evaluate the optimum roughage to concentrate ratio in maize stover (MS) based complete diets for efficient microbial biomass production (EMBP) using *in vitro* gas production technique.

**Materials and Methods:**

MS based complete diets with roughage to concentrate ratio of 100:0, 90:10, 80:20, 70:30, 60:40, 50:50, 40:60, and 30:70 were formulated, and 200 mg of oven-dried sample was incubated in water bath at 39°C along with media (rumen liquor [RL] - buffer) in *in vitro* gas syringes to evaluate the gas production. The gas produced was recorded at 8 and 24 h of incubation. *In vitro* organic matter digestibility (IVOMD), metabolizable energy (ME), truly digestible organic matter (TDOM), partitioning factor (PF), and EMBP were calculated using appropriate formulae. Ammonia nitrogen and total volatile fatty acids (TVFAs) production were analyzed in RL fluid-media mixture after 24 h of incubation.

**Results:**

*In vitro* gas production (ml) at 24 h incubation, IVOMD, ME, TDOM, TVFA concentration, and ammonia nitrogen production were increased (p<0.01) in proportion to the increase in the level of concentrate in the diet. Significantly (p<0.01) higher PF and EMBP was noticed in total mixed ration with roughage to concentrate ratio of 60:40 and 50:50 followed by 70:30 and 40:60.

**Conclusion:**

Based on the results, it was concluded that the MS can be included in complete rations for ruminants at the level of 50-60% for better microbial biomass synthesis which in turn influences the performance of growing sheep.

## Introduction

The livestock industry is an important sector in the developing countries of Asia and Africa, and it provides income for small-scale farmers in addition to those generated from agriculture. Although the livestock population is high in developing countries, the production level per head is lower compared to developed countries. This is mainly due to shrinkage of grazing land which indirectly affects the ability of small and marginal scale farmers to feed animals adequately throughout the year [1]. To overcome such problems, we need to develop another alternate economic feeding system. Incorporation of locally available crop residues (CR), such as cereal straws and stovers, in the form of the complete ration, is an alternative plausible option for ruminant feeding because the blend of unpalatable roughages and concentrates offers little choice to the animal selection of specific ingredients in feed [2]. Moreover, optimization of roughage (R) to concentrate (C) ratio (R:C) improves the efficiency of nutrient utilization. In addition to this, an optimum level of roughage supplementation is essential in ruminant diets to maintain the desirable volatile fatty acids (VFA) production pattern.

Cereal CR are an important feed source for ruminants in smallholders crop-livestock system in the (sub) tropics [3] contributing about 74% of worldwide total CR [4]. Furthermore, CR need no additional allocation of water and land because they are byproducts of crops primarily grown for their principal product. Maize or corn (*Zea mays*) is the most producing crop in Asia and Africa after rice (paddy) and wheat [3]. Maize stover (MS) is estimated to be a contributor over a third of cereal CR production at the global level and for Africa as a whole, but more than 60% is in Latin America and 20% in Asia [3]. Maize is currently produced on nearly 100 million ha in 125 developing countries and is among the three most widely grown crops in 75 of those countries [5]. At the global level, 63% of the demand for maize grain is now used for livestock feed to the extent of 70% in developed countries and 56% in developing countries [6]. In Asia and Africa, maize is the most preferred cereal grain source in poultry diet (60-70%) due to its high energy content and good palatability. The increasing demand for maize grain for feeding pig and poultry is at least regionally matched by increasing demand for MS as ruminant feedstuff [3,7]. In addition to this, price fluctuations in sorghum stover (a conventional roughage source) [8] increased the importance of MS inclusion as roughage source in ruminant diets.

Therefore, the present study was undertaken to optimize the MS to concentrate ratios in MS based completed diets for efficient microbial biomass production (EMBP) using *in vitro* gas production technique so as to recommend to the smallholder livestock farmers for suitable production.

## Materials and Methods

### Ethical approval

Experimental animals were handled as outlined by guidelines of Institutional Animal Ethics Committee, College of Veterinary Science, Hyderabad, and Sri Venkateswara Veterinary University.

### Sample preparation

MS and concentrate mixture (Table-1) were ground using cyclone mill having 1 mm screen. Experimental complete diets with roughage to concentrate ratio of 100:0, 90:10, 80:20, 70:30, 60:40, 50:50, 40:60, and 30:70 were formulated and 200 mg of oven dried experimental complete diet samples were weighed in triplicate for *in vitro* gas evaluation. The chemical composition of maize stove and concentrate mixture are presented in Table-2.

**Table-1:** Ingredient composition of the concentrate mixture.

Ingredient	%
Maize	31.0
Groundnut cake	16.5
Sunflower cake	20.0
Deoiled rice bran	23.0
Molasses	5.0
Urea	1.5
Mineral mixture	2.0
Salt	1.0

**Table-2:** Chemical composition (% DM basis) of experimental feeds and complete diets.

Parameter	Maize stover (%)	Concentrate mixture (%)
DM	90.5	89.5
Organic matter	88.59	89.65
Crude protein	5.51	20.52
Crude fiber	30.99	16.89
Ether extract	2.39	1.82
Nitrogen free extract	49.69	49.33
Total ash	11.41	10.35
NDF	77.93	27.19
Acid detergent fiber	43.23	18.21
Hemicellulose	34.72	9.32
Cellulose	35.83	13.51
Lignin	6.51	3.89

Each value is average of three observations. NDF=Neutral detergent fiber, DM=Dry matter

### Collection of rumen liquor (RL) and media preparation

The media used for *in vitro* gas production technique was prepared using various solutions (distilled water - 10 ml; macro mineral solution - 5 ml; bicarbonate buffer - 5 ml; micro mineral solution - 0.25 µl; resazurin solution - 0.025 ml, and reduction solution - 0.06 ml) [9]. RL was collected from three Deccani rams using stomach tube fitted with a vacuum pump before offering morning feed. These animals were fed on chopped MS and supplemented concentrate mixture. Approximately 350 ml RL was collected from different depths and directions of the reticulorumen in a pre-warmed thermos flask and transferred to the laboratory and strained through pre-warmed (kept in incubator for overnight at 39°C) 4-fold muslin cloth and flushed with CO_2_. Rumen fluid-buffer media mixture prepared by mixing RL (10 ml) and bicarbonate mineral distilled water mixture (20 ml) under continuous flushing with CO_2_, then this media mixture was injected into pre-warmed (39°C) glass syringes (Fortuna Optima, Germany) containing 200 mg sample, after addition of media, glass syringes were immediately transferred into water bath (39°C) and incubated for 24 h.

### Measurement of *in vitro* parameters

The gas production was recorded at 8 and 24 h of incubation. After corrected for blank and standard, the gas production at 24 h was used to determine the metabolizable energy (ME) [6] and *in vitro* organic matter digestibility (IVOMD) [10]. Truly digestible organic matter (TDOM) is estimated by refluxing the residue of substrate left in the syringe after termination of incubation (24 h) with a neutral detergent solution and estimating neutral detergent fiber (NDF) in the undigested residue. The difference between the organic matter in the substrate before incubation and NDF in the undigested residue gives TDOM. The ratio of TDOM and gas volume produced (24 h) was used to know the partitioning factor (PF) [11]. The MBP and EMBP were calculated as described by Blümmel *et al*. [12] considering a stoichiometric factor 2.20. Ammonia nitrogen [13] and total VFA (TVFAs) [14] production were analyzed in RL fluid-media mixture after 24 h of incubation. Feed samples were analyzed for proximate principles [15] and fiber fractions [16].

### Statistical analysis

The data obtained in this study were subjected to one-way analysis of variance. The differences between the means were tested for significance using Duncan’s multiple range test [17]. All the statistical procedures carried out using Statistical Package for Social Sciences 16^th^ version.

## Results

*In vitro* gas production, IVOMD, TDOM, and ME values were increased (p<0.01) linearly as increasing the proportion of concentrate (0-70%) in complete diets (Table-3). Similarly, concentrate inclusion in complete diets increased (p<0.01) the TVFA (mEq/L) (Table-3) and rumen ammonia nitrogen production (mg/100 ml) (Table-4). Whereas, highest (p<0.01) PF, MBP, and EMBP were noticed at 60R:40C and 50R:50C (Table-4) ratio compared to other combinations.

**Table-3:** Effect of MS to concentrate ratio on *in vitro* gas production parameters in Deccani sheep.

Roughage:Concentrate	Gas volume (ml/200 mg)	IVOMD (mg)	ME (MJ/kg DM)	TDOM (mg)	TVFA (mEq/L)
100:0	43.00^g^	94.60^g^	8.15^d^	123.53^g^	26.33^c^
90:10	44.00^ef^	96.80^ef^	8.32^cd^	131.86^f^	26.00^c^
80:20	45.00^ef^	99.00^ef^	8.50^cd^	140.70^e^	26.00^c^
70:30	45.83^de^	100.83^de^	8.62^bcd^	146.09^d^	31.33^b^
60:40	47.50^cd^	104.50^cd^	8.85^bc^	153.51^c^	37.67^a^
50:50	48.17^bc^	105.97^bc^	9.00^abc^	154.42^c^	40.33^a^
40:60	49.83^b^	109.63^b^	9.26^ab^	157.78^b^	36.00^a^
30:70	52.50^a^	115.50^a^	9.62^a^	160.67^a^	37.00^a^
SEM	0.65	1.42	0.12	2.58	1.52

Values bearing different superscripts in a column differ significantly (p<0.01). IVOMD=*In vitro* organic matter digestibility, ME=Metabolizable energy, TDOM=Truly digestible organic matter, TVFA=Total volatile fatty acids

**Table-4:** Effect of MS to concentrate ratio on PF, EMBP, and ammonia-nitrogen in Deccani sheep.

Roughage:Concentrate	Ammonia N (mg/100 ml)	PF	MBP (mg)	EMBP (%)
100:0	17.07^g^	2.87^d^	28.93^d^	23.43^d^
90:10	22.40^f^	3.00^cd^	35.06^c^	26.59^c^
80:20	25.87^e^	3.13^abc^	41.70^b^	29.62^ab^
70:30	30.13^d^	3.19^ab^	45.25^ab^	30.98^ab^
60:40	32.53^d^	3.23^a^	49.01^a^	31.92^a^
50:50	40.00^c^	3.21^a^	48.45^a^	31.38^a^
40:60	45.87^b^	3.17^ab^	48.15^a^	30.53^ab^
30:70	56.00^a^	3.06^bc^	45.17^ab^	28.11^bc^
SEM	2.53	0.02	1.47	0.63

Values bearing different superscripts in a column differ significantly (p<0.01). PF=Partitioning factor, MBP=Microbial biomass production, EMBP=Efficiency of microbial biomass production, SEM=Standard error of mean, MS=Maize stover

## Discussion

### Gas production and organic matter digestibility

Gas production (ml/200 mg), which reflects the apparent substrate degradability [18], was increased (p<0.01) linearly in accordance with the proportion of concentrate (0-70%) in the inoculum. Significantly (p<0.01) highest and lowest gas production was recorded at 30R:70C and 100R:0C, respectively (Table-3). This increase in gas production with increased concentrate proportion in the inoculum, might be due to increase in the nutrient availability from concentrate [19] or might be due to lowered cell wall and lignin content in the inoculum as reduction in roughage proportion in the inoculum, which negatively affect the microbial attachment to the feed particles [20,21]. Similar to our results, previous researchers [22-24] also observed an increase in gas volume as a proportion of roughage was replaced by concentrate in the complete ration.

Similar to gas production, IVOMD, TDOM, and ME values were increased linearly as the concentrate proportion increased from 0% to 70% (Table-3). The increased values might be due to gradual reduction in hemicellulose, cellulose, and lignin content in inoculum from 100R:0C to 30R:70C which act as limiting factors to lower the digestibility at excess amount [20,21] or increased nutrient availability to microorganisms from increased proportion of concentrate in the ration [19]. Similarly, Polyorach *et al*. [25] observed an increase in IVOMD in complete rations by increasing the concentrate proportion from 20% to 80%. The results of the present study were also in agreement with Blümmel *et al*. [26] and Khanum *et al*. [27], who stated that CR digestibility can be improved when CR are supplemented with concentrate which provides better nutrients to microorganisms than roughage alone. Similarly, TVFA (mEq/L) production was also increased (p<0.01) (Table-3) as the level of concentrate increased in the complete diet. This result is inconsistent with the findings of Getachew *et al*. [28], who concluded that TVFA production was positively correlated (p<0.01) with *in vitro* gas production.

### Ammonia production and microbial biomass synthesis

The rumen ammonia nitrogen concentration increased (p<0.01) linearly as increasing the concentrate proportion in the complete diet. Significantly (p<0.01) highest and lowest ammonia nitrogen concentration was noticed with the rations containing 70% and 0% concentrates. This ammonia nitrogen increased in proportion to the concentrate in complete diet might be due to active degradation of protein and hydrolysis of non-protein nitrogen substances. Similar to our results, Kumari *et al*. [22] and Reddy *et al*. [24] observed increase in ammonia nitrogen concentration in complete diets having more concentrate proportion. During incubation, the liberated ammonia will be incorporated into microbial protein synthesis, but this incorporation depends on synchronization between availability of nitrogen and energy [29]. In the current study, though the ammonia nitrogen was the highest at 30R:70C, but the highest MBP synthesis was recorded at 60R:40C, 50R:50C, and 40R:60C (Table-4). This might be due to synchronization between liberated ammonia and availability of fermentable carbohydrates to the rumen bacteria. Similar to our results, Kumari *et al*. [22] and Reddy *et al*. [24] observed higher (p<0.01) MBP synthesis with 60R:40C, 50R:50C in complete diets though they noticed the highest ammonia production at 30R:70C. PF is an index for distribution of substrate truly degraded between microbial mass and fermentation products [30]. Therefore, PF provides meaningful information for prediction of MBP and also voluntary intake in ruminants. Similarly, Thirumalesh and Krishnamoorthy [30] noticed a positive correlation between microbial biomass flow to duodenum and PF of the total mixed ration. In the present investigation, higher (p<0.01) PF was noticed at 60R:40C, 50R:50C followed by at 70R:30C and 40R:60C. The higher EMBP was also observed at 60R:40C, 50R:50C followed by at 70R:30C and 40R:60C, which was positively correlated with PF of the ration as has been reported previously [22,24].

## Conclusion

Based on the results, it was concluded that MS can be included between 50% and 60% level in complete rations for the economic rearing of ruminant animals.

## Authors’ Contributions

YRR and NNK implemented the study design. YRR, NNK, TM, and KS recorded the data and analyzed. NNK and KS drafted the manuscript. YRR, NNK, TM, and KS revised the manuscript. All authors read and approved the final manuscript.
